# Hepatitis C Virus (HCV) Genotype 1 Subtype Identification in New HCV Drug Development and Future Clinical Practice

**DOI:** 10.1371/journal.pone.0008209

**Published:** 2009-12-08

**Authors:** Stéphane Chevaliez, Magali Bouvier-Alias, Rozenn Brillet, Jean-Michel Pawlotsky

**Affiliations:** 1 French National Reference Center for Viral Hepatitis B, C and delta, Department of Virology, Hôpital Henri Mondor, Université Paris 12, Créteil, France; 2 INSERM U955, Créteil, France; Yale University, United States of America

## Abstract

**Background:**

With the development of new specific inhibitors of hepatitis C virus (HCV) enzymes and functions that may yield different antiviral responses and resistance profiles according to the HCV subtype, correct HCV genotype 1 subtype identification is mandatory in clinical trials for stratification and interpretation purposes and will likely become necessary in future clinical practice. The goal of this study was to identify the appropriate molecular tool(s) for accurate HCV genotype 1 subtype determination.

**Methodology/Principal Findings:**

A large cohort of 500 treatment-naïve patients eligible for HCV drug trials and infected with either subtype 1a or 1b was studied. Methods based on the sole analysis of the 5′ non-coding region (5′NCR) by sequence analysis or reverse hybridization failed to correctly identify HCV subtype 1a in 22.8%–29.5% of cases, and HCV subtype 1b in 9.5%–8.7% of cases. Natural polymorphisms at positions 107, 204 and/or 243 were responsible for mis-subtyping with these methods. A real-time PCR method using genotype- and subtype-specific primers and probes located in both the 5′NCR and the NS5B-coding region failed to correctly identify HCV genotype 1 subtype in approximately 10% of cases. The second-generation line probe assay, a reverse hybridization assay that uses probes targeting both the 5′NCR and core-coding region, correctly identified HCV subtypes 1a and 1b in more than 99% of cases.

**Conclusions/Significance:**

In the context of new HCV drug development, HCV genotyping methods based on the exclusive analysis of the 5′NCR should be avoided. The second-generation line probe assay is currently the best commercial assay for determination of HCV genotype 1 subtypes 1a and 1b in clinical trials and practice.

## Introduction

Over 170 million individuals are infected with hepatitis C virus (HCV) worldwide. Phylogenetic analyses have shown that HCV strains can be classified into at least 6 major genotypes (numbered 1 to 6), and a large number of subtypes within each genotype [1]. Genotype 1 is by far the most frequent genotype in chronically infected patients worldwide, with subtypes 1a and 1b representing the vast majority of circulating strains [2], [3], [4].

Current treatment of chronic hepatitis C is based on the combination of pegylated interferon (IFN)-α and ribavirin [5]. This treatment fails to eradicate infection in 50%–60% of patients infected with HCV genotype 1 and approximately 20% of those infected with HCV genotypes 2 and 3 [6], [7], [8]. Thus the need for more efficacious therapies is urgent, especially for patients infected with HCV genotype 1. A number of novel antiviral molecules currently are in preclinical or clinical development [9]. The most advanced ones are specific inhibitors of viral enzymes and functions involved in the HCV life cycle. Molecules that have reached clinical development include inhibitors of the nonstructural (NS) 3/4A serine protease and inhibitors of HCV replication that belong to different categories: nucleoside/nucleotide analogue and non-nucleoside inhibitors of the HCV RNA-dependent RNA polymerase (RdRp), NS5A inhibitors and cyclophilin inhibitors [9]. These agents have shown potent antiviral efficacy when used alone, and encouraging results have been recently published showing that HCV clearance can be achieved in approximately 70% of cases when a potent NS3/4A inhibitor is used in combination with pegylated IFN-α and ribavirin [10], [11], [12].

HCV genotype 1 is generally considered as a homogeneous group. There are however biological differences between the different subtypes of HCV genotype 1, which are related to differences in their nucleotide and amino acid sequences. Importantly, differences between subtype 1a and 1b (by far the most frequently encountered genotype 1 subtypes in clinical practice) include different efficacies of antiviral drugs and different resistance profiles to such drugs. Indeed, several HCV inhibitors appear to have selective activity against different HCV genotype 1 subtypes, both *in vitro* and *in vivo*. Differences have been observed *in vitro* with NS3/4A protease inhibitors, non-nucleoside inhibitors of HCV RdRp and NS5A inhibitors [13], [14], [15], [16], [17]. For instance, BILB 1941, a non-nucleoside inhibitor of HCV RdRp, has been shown to have better antiviral efficacy in patients infected with HCV subtype 1b than in those infected with HCV subtype 1a, a finding reflecting *in vitro* experiments [13].

A major issue that limits the efficacy of direct acting antiviral therapies for HCV is the selection by these drugs of resistant variants upon administration [18]. Recent studies with NS3/4A protease inhibitors have shown that the genetic barrier and resistance profiles substantially differ between the different genotype 1 subtypes. For instance, the Arg to Lys substitution at position 155 of the NS3 protease (R155K) is usually selected in subtype 1a replicons treated with telaprevir, but not in subtype 1b replicons [19]. The reason is that only one nucleotide substitution is needed relative to the subtype 1a sequence to generate this variant, whereas two substitutions are needed relative to the 1b sequence (codon usage bias). Overall, natural polymorphisms at positions R155 and V36 are frequent in subtype 1a, but rare in subtype 1b where substitutions at position A156 are preferentially selected *in vitro* [19]. This is reflected *in vivo* by the different resistance profiles in patients infected by HCV subtypes 1a and 1b. In the former, the V36 and R155 substitutions represent the backbone of resistance, whereas in the latter resistance is less frequent as it is preferentially associated with substitutions at position A156 that are associated with a decreased fitness of the variants [19], [20], [21]. Similarly, important differences in the resistance profiles have been described *in vitro* with HCV-796, a non-nucleoside inhibitor of HCV RdRp. The C316Y amino acid substitution has been reported to be selected in both subtype 1a and 1b replicon cells. However, in genotype 1a replicons, the C316Y substitution has low replication capacity that must be compensated for by additional “compensatory” substitutions, including L392F or M414T, resulting in an increase in replication levels of at least 10-fold [19]. A higher genetic barrier to resistance to HCV-796 and related compounds is therefore expected in patients infected with HCV subtype 1a than 1b. *In vivo*, HCV-796 monotherapy was however shown to select subtype 1a variants with a single C316Y substitution, whereas the C316Y substitution was associated with a number of additional substitutions in subtype 1b patients [22].

As a result of these findings, correct identification of HCV subtypes 1a and 1b is crucial in clinical trials assessing new HCV drugs in order to correctly stratify and interpret efficacy and resistance data. It may also become important in future clinical practice, as tailoring treatment schedules with HCV inhibitors to HCV genotype 1 subtype might become necessary. A variety of molecular methods can be used to identify the HCV genotype and subtype both in clinical trials and practice. Commercial assays have been developed, most of them targeting the 5′ noncoding region (5′NCR) of the HCV genome, although this region is the most conserved one. These methods have been shown to differentiate well the different HCV genotypes (1 to 6), except genotype 1 from genotype 6, a rare HCV genotype in the Western world [23], [24]. The goal of our study was to assess the ability of molecular methods targeting the 5′NCR to correctly identify the HCV genotype 1 subtype in patients eligible for clinical trials, and to identify the best method for this purpose.

## Results

### Hepatitis C Virus Genotype and Subtype Determination by Phylogenetic Analysis of a Portion of the NS5B Gene

**Figure 1:**
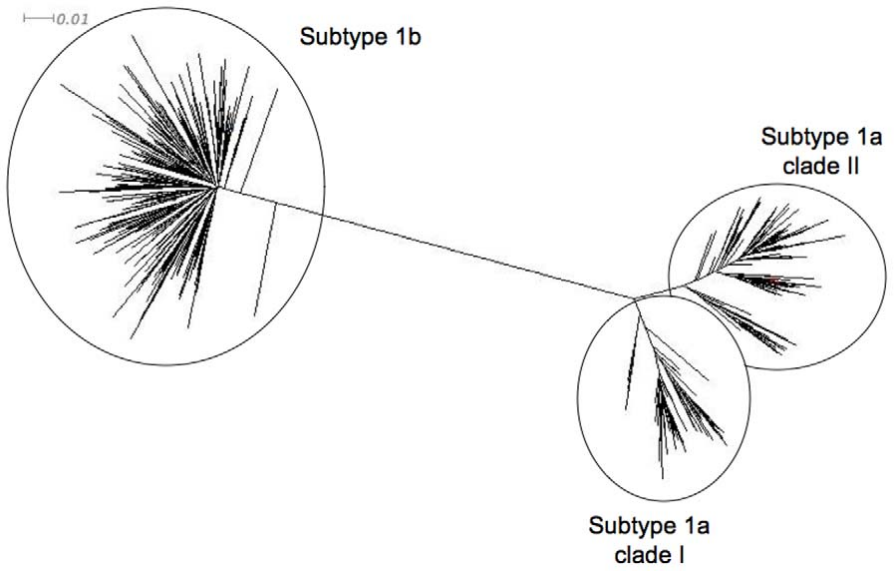
Phylogenetic tree plotted with NS5B sequences (nucleotide positions 8325-8610) from the 237 HCV subtype 1a and 263 HCV subtype 1b strains. HCV subtype 1a strains segregated into two distinct clades, termed 1a clade I and 1a clade II.

Direct sequence analysis of a sufficiently long portion of the NS5B gene followed by phylogenetic analysis is the reference method for identification of HCV genotype and subtype [1], [25]. It was used to identify the HCV genotype and subtype in 516 treatment-naïve patients included in a multicenter clinical trial assessing different schedules of pegylated IFN-α2a and ribavirin [26]. All of these patients were thought to be infected with HCV genotype 1 at inclusion based on local assessment. In fact, 6 patients were infected with genotype 6, including 2 with subtype 6e, one with subtype 6o, one with subtype 6p, one with subtype 6q and one with subtype 6r. These 6 samples were not considered for further analysis in the present study. The remaining 510 patients were confirmed to be infected with HCV genotype 1: 237 of them (46.5%) were infected with HCV subtype 1a and 263 (51.6%) with subtype 1b (Figure 1). As shown in Figure 1, HCV subtype 1a strains segregated into two distinct clades, that were termed 1a clade I (n = 83, 35.0%) and 1a clade II (n = 154, 65.0%). Eight patients (1.6%) were infected with another HCV genotype 1 subtype, including 4 patients with subtype 1d, 2 with subtype 1e, one with subtype 1i, and one with subtype 1l. The remaining 2 patients (0.3%) were infected with genotype 1 but the subtype could not be determined. The ability of the different molecular methods to correctly identify HCV subtypes 1a and 1b was then tested on the 237 and 263 samples containing HCV subtypes 1a and 1b, respectively.

### Ability of Commercial HCV Genotype/Subtype Determination Methods to Correctly Identify HCV Subtypes 1a and 1b

Table 1 shows the proportion of HCV subtype 1a and 1b samples that were correctly identified by the molecular methods tested in this study. The results are shown globally, and after removing the samples that could not be amplified with the PCR technique used in the assay.

**Table 1:** Ability of the different molecular methods tested in this study to correctly identify HCV subtypes 1a and 1b in a series of 500 patients infected by one or the other of these subtypes.

Assay		Trugene HCV 5′NC Genotyping Assay	INNO-LiPA HCV 1.0	INNO-LiPA HCV 2.0	Abbott RealTi*m*e HCV Genotype II assay
Manufacturer		Siemens Medical Solutions Diagnostic	Innogenetics	Innogenetics	Abbott Molecular
Method		**Sequence analysis of the 5′NCR followed by sequence comparison**	**Reverse hybridization targeting the 5′NCR**	**Reverse hybridization targeting the 5′NCR and the core-coding region**	**Real-time PCR assay targeting the 5′NCR and NS5B-coding region**
**All samples**	**Subtype 1a** * **(N = 237), n/N (%)**	183/237 (77.2%)	167/237 (70.5%)	231/237 (97.5%)	220/236** (93.2%)
	**Subtype 1b** * **(N = 263), n/N (%)**	238/263 (90.5%)	240/263 (91.3%)	253/263 (96.2%)	232/261** (88.9%)
**Samples that could be PCR-amplified only**	**Subtype 1a** * **, n/N (%)**	183/235(77.9%)	167/236 (70.8%)	231/232 (99.6%)	220/236 (93.2%)
	**Subtype 1b** * **, n/N (%)**	238/258 (92.2%)	240/260 (92.3%)	253/255 (99.2%)	232/259 (89.6%)

Correct identification with the different techniques tested is shown for all samples, and for samples that could be amplified by PCR in the assay.

* The correct HCV genotype 1 subtype was identified by means of direct sequence analysis of a portion of the NS5B gene followed by phylogenetic analysis, the reference method.

** In one 1a case and two 1b cases, not enough serum volume was available for testing in the Abbott RealTi*m*e HCV Genotype II assay.

Methods based on the sole analysis of the 5′NCR, namely Trugene HCV Genotyping Kit and INNO-LiPA HCV 1.0, failed to correctly identify HCV subtype 1a in 22.8% and 29.5% of cases, and HCV subtype 1b in 9.5% and 8.7% of cases, respectively (Table 1). Only 7 and 4 samples, respectively, could not be PCR-amplified by these methods. Thus, the failure to correctly identify the HCV subtype was due to erroneous classification in the vast majority of these cases (Table 1). Two (2.5%) and 14 (17.3%) of the 81 strains belonging to subtype 1a clade I that could be PCR-amplified were incorrectly subtyped by Trugene HCV Genotyping Kit and INNO-LiPA HCV 1.0, respectively. On the other hand, 50 (32.7%) and 55 (35.9%) of the 153 strains belonging to subtype 1a clade II that could be PCR-amplified were incorrectly classified by these two methods, respectively. Table 2 shows the results given by the two methods targeting the 5′NCR only, Trugene HCV Genotyping Kit and INNO-LiPA HCV 1.0, in the samples that were not correctly classified as either 1a or 1b by these methods.

**Table 2:** Number of samples displaying discrepancies between the assays targeting the 5′NCR only and the reference method in the 500 samples infected with either subtype 1a or subtype 1b.

Result with the reference method	Result with the 5′NCR targeting method	Trugene HCV 5′NC Genotyping Assay	INNO-LiPA HCV 1.0
HCV subtype 1a	Subtype 1b Subtype 1a or 1b* Genotype 1, indeterminate subtype Genotype 1, other subtype Other genotype Result not interpretable Negative PCR amplification	40 0 12 0 0 0 2	40 4 25 0 0 0 1
HCV subtype 1b	Subtype 1a Subtype 1a or 1b* Genotype 1, indeterminate subtype Genotype 1, other subtype Other genotype Result not interpretable Negative PCR amplification	7 0 11 0 1 1 5	4 1 15 0 0 0 3

* The assay has been unable to differentiate between subtypes 1a and 1b.

INNO-LiPA HCV 2.0 displays the same 5′NCR oligonucleotide probes as INNO-LiPA HCV 1.0, plus core-encoded oligonucleotide probes aimed at better discriminating between HCV subtypes 1a and 1b. With INNO-LiPA HCV 2.0, subtype identification was corrected in 64 of the 70 subtypes 1a that were incorrectly typed with INNO-LiPA HCV 1.0. Five samples could not be PCR-amplified in the core-coding region and the result was not interpretable with INNO-LiPA HCV 2.0 in the remaining case (Table 1). INNO-LiPA HCV 2.0 also corrected subtype identification in 13 of 23 subtypes 1b that were incorrectly typed with INNO-LiPA HCV 1.0. Eight samples could not be PCR-amplified in the core-coding region and the result was not interpretable with INNO-LiPA HCV 2.0 in the remaining two cases (Table 1). Overall, the second-generation line probe assay correctly classified 97.5% of subtype 1a and 96.2% of subtype 1b strains. When only samples that could be PCR-amplified with the assay procedure were taken into account, correct subtype determination was achieved in 99.6% and 99.2% of cases, respectively (Table 1).

The real-time PCR-based assay targeting both the 5′NCR and the NS5B region, Abbott RealTi*m*e HCV Genotype II assay, correctly identified 93.2% of subtype 1a and 88.9% of subtype 1b strains. Only 2 HCV subtype 1b samples could not be PCR-amplified with this method (Table 1).

### 5′NCR Sequence Analysis in Misclassified Subtype 1a Strains

**Figure 2:**
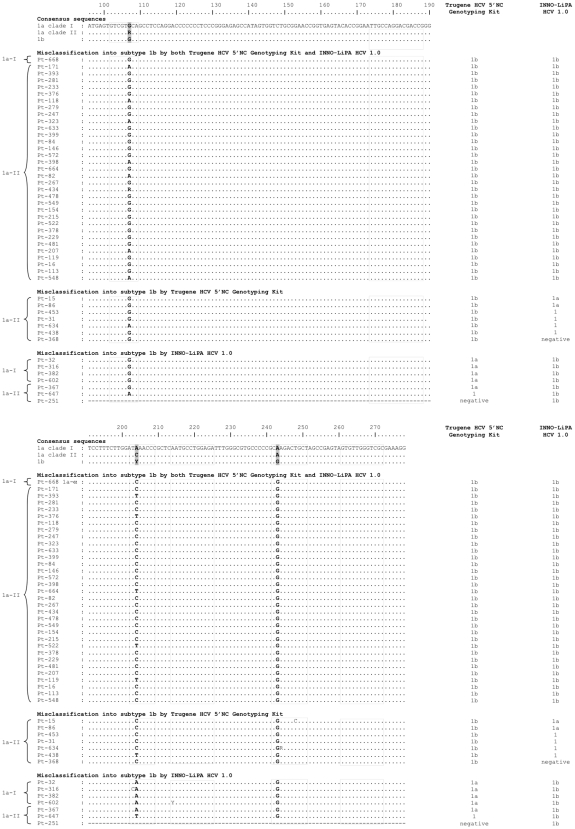
Alignment of the 5′NCR sequences from the subtype 1a strains that were incorrectly classified by Trugene HCV Genotyping Kit and/or INNO-LiPA HCV 1.0 relative to the consensus sequences of the correctly classified strains, including subtype 1a clade I (1a-I), subtype 1a clade II (1a-II) and subtype 1b. Positions 107, 204 and 243, that differentiate subtypes 1a and 1b are in bold. The dotted squares represent the location of the INNO-LiPA HCV 1.0 oligonucleotide probes. The result given by each assay is shown on the right.

Among the HCV subtype 1a strains, 47 were misclassified as subtype 1b by Trugene HCV Genotyping Kit and/or INNO-LiPA HCV 1.0, including 33 that were misclassified by both assays, 7 that were misclassified by Trugene HCV Genotyping Kit only, and 7 that were misclassified by INNO-LiPA HCV 1.0 only (Table 2). Figure 2 shows an alignment of their 5′NCR sequences relative to the consensus sequences of the correctly classified strains (including subtype 1a clade I, subtype 1a clade II and subtype 1b). As shown in Figure 2, misclassification of subtype 1a strains into subtype 1b in one or both assays was related to the presence of natural polymorphisms at nucleotide positions 204 and 243, both of which are located within the sequence of an INNO-LiPA HCV 1.0 probe. At position 243, A is the most frequent nucleotide in HCV subtype 1a, in both subtype 1a clade I and clade II. Substitution into a G, the most frequent nucleotide at position 243 in subtype 1b, was found in all cases that were misclassified as subtype 1b by Trugene HCV Genotyping Kit and/or INNO-LiPA HCV 1.0 (Figure 2). At position 204, A is the most frequent nucleotide for subtype 1a clade I, whereas C is the most frequent nucleotide for subtype 1a clade II, and C or T are the most frequent nucleotides for subtype 1b. In spite of the presence of a G at position 243, the presence of an A at position 204 allowed correct identification of subtype 1a with Trugene HCV Genotyping Kit but not with INNO-LiPA HCV 1.0 (Figure 2). The usual presence of a C at position 204 in subtype 1a clade II explains why misclassifications were far more frequent with this clade than with subtype 1a clade I.

Among the 12 subtype 1a strains that were classified as genotype 1, indeterminate subtype with Trugene HCV Genotyping Kit, one had a G and 5 had mixed A and G populations at position 243. Two additional patients with an A at position 243 had a C at position 248. In the remaining 4 cases, no explanation was found in the 5′NCR sequence for the failure to identify the HCV subtype (data not shown). Among the 25 subtype 1a strains that were classified as genotype 1, indeterminate subtype with INNO-LiPA HCV 1.0 (including 6 with the same profile in Trugene HCV Genotyping Kit), 4 had a G and 4 had mixed A and G populations at position 243. Three additional patients with an A at position 243 had a C at position 248 (C only in two of them, a mixture of C and T in one). In the 14 remaining cases, no explanation was found in the 5′NCR sequence for the failure to identify the HCV subtype (data not shown).

### 5′NCR Sequence Analysis in Misclassified Subtype 1b Strains

**Figure 3:**
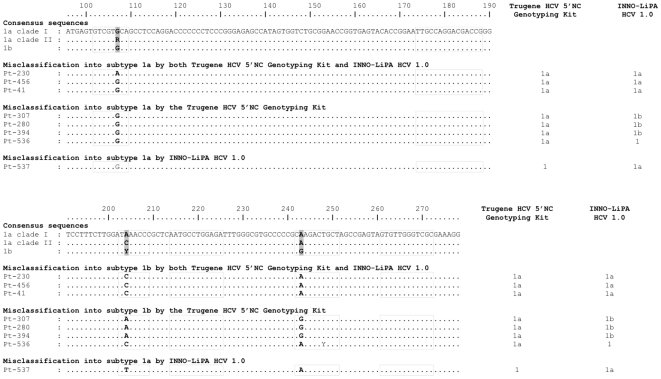
Alignment of the 5′NCR sequences from the subtype 1b strains that were incorrectly classified by Trugene HCV Genotyping Kit and/or INNO-LiPA HCV 1.0 relative to the consensus sequences of the correctly classified strains, including subtype 1a clade I, subtype 1a clade II and subtype 1b. Positions 107, 204 and 243, that differentiate subtypes 1a and 1b are in bold. The dotted squares represent the location of the INNO-LiPA HCV 1.0 oligonucleotide probes. The result given by each assay is shown on the right.

Among HCV subtype 1b strains, 8 were misclassified as subtype 1a by Trugene HCV Genotyping Kit and/or INNO-LiPA HCV 1.0, including 3 that were misclassified by both assays, 4 that were misclassified by Trugene HCV Genotyping Kit only, and 1 that was misclassified by INNO-LiPA HCV 1.0 only (Table 2). Figure 3 shows an alignment of their 5′NCR sequences relative to the consensus sequences of the correctly classified subtype 1a and subtype 1b strains. As shown in Figure 3, and as for misclassified subtype 1a strains discussed above, misclassification of subtype 1b strains into subtype 1a was related to the presence of natural polymorphisms at positions 204 and 243. At position 243, G is the most frequent nucleotide in HCV subtype 1b. Substitution into an A, the most frequent nucleotide at position 243 in subtype 1a, was found in all cases that were misclassified as subtype 1a by both Trugene HCV Genotyping Kit and INNO-LiPA HCV 1.0 and by INNO-LiPA HCV 1.0 only, but not in those that were misclassified by Trugene HCV Genotyping Kit only (Figure 3). In the latter, it is the presence of an A at position 204 instead of a C or a T that was responsible for misclassification in all but one case (Figure 3).

Among the 11 subtype 1b strains that were classified as genotype 1, indeterminate subtype with Trugene HCV Genotyping Kit, one had an A at position 243. In the remaining cases, no explanation was found in the 5′NCR sequence for the failure to identify the HCV subtype (data not shown). Among the 15 subtype 1b strains that were classified as genotype 1, indeterminate subtype with INNO-LiPA HCV 1.0 (none of which were classified as indeterminate in Trugene HCV Genotyping Kit), one had an A and one harbored mixed A and G populations at position 243. Both of them had a C at position 248 (C only in one of them and a mixture of C and T in the other one). In the remaining 13 cases, no explanation was found in the 5′NCR sequence for the failure to identify the HCV subtype (data not shown).

### Incorrect Subtyping with Abbott RealTi*m*e HCV Genotype II Assay, that Targets Both the 5′NCR and NS5B Region

Among the HCV subtype 1a strains, 16 were incorrectly classified by Abbott RealTi*m*e HCV Genotype II assay (Table 1): 2 were misclassified as subtype 1b, 12 were classified as genotype 1, indeterminate subtype, one was identified as a mixed 1a/1b infection, and one gave an indeterminate result. In one case, PCR amplification failed, and in one case, not enough serum volume was available for testing.

Among the HCV subtype 1b strains, 27 were incorrectly classified by Abbott RealTi*m*e HCV Genotype II assay (Table 1): 3 were misclassified as subtype 1a, 18 were classified as genotype 1, indeterminate subtype, 5 were identified as a mixed 1a/1b infection, and one gave an indeterminate result. In 2 cases, PCR amplification failed, and in one case, not enough serum volume was available for testing.

## Discussion

HCV genotype determination is needed in clinical practice to decide the dose of ribavirin and the duration of pegylated IFN-α-ribavirin treatment [5]. In contrast, subtype identification has no clinical impact on current therapy. However, this is changing with the development of new specific inhibitors of HCV enzymes and functions that may yield different antiviral responses and resistance profiles according to the HCV genotype subtype [13], [14], [15], [16], [17], [18], [19], [20], [21], [22]. Correct HCV genotype 1 subtype identification is mandatory in clinical trials where these drugs are tested alone, in combination, or in combination with pegylated IFN-α and ribavirin for stratification and interpretation purposes. It might also become necessary in future clinical practice when several of these drugs have reached the market and a number of treatment choices are available for HCV-infected patients.

In this context, the goal of our study was to identify the appropriate molecular tool(s) for accurate HCV genotype 1 subtype determination in clinical trials and future practice. Our study was performed in a large cohort of 500 treatment-naïve patients eligible for HCV drug clinical trials and infected with either subtype 1a or 1b, based on the reference method, i.e. direct sequence analysis of the NS5B region followed by phylogenetic analysis [1], [27]. The results clearly show that, although they are by far the most widely used techniques in new HCV drug development trials, genotyping techniques based on the sole analysis of the 5′NCR should be avoided, as they mistype approximately 25% and 10% of HCV subtype 1a and 1b strains, respectively.

The accuracy of genotype and subtype determination depends on the amount of information (i.e. the number of informative sites) that is utilized for discrimination in the tested region. Our results show that only three positions in the 5′NCR can be used to discriminate HCV subtypes 1a and 1b, including positions 107, 204 and 243, and that natural polymorphisms at these positions are responsible for mis-subtyping with methods analyzing exclusively the 5′NCR. Indeed, all of these positions are located within the sequence of INNO-LiPA HCV 1.0 oligonucleotide probes and they are used in the sequence comparison algorithms of Trugene HCV Genotyping Kit. The presence of a substitution at position 243 has already been reported to yield mistyping with methods targeting the 5′NCR [28], [29]. We show here that polymorphisms at position 204 also play a role. Additional changes at position 248 could also play a role in mis-subtyping as they were observed in several patients who had no polymorphism at position 243. In addition, analysis of the 5′NCR has been reported not to discriminate well between HCV genotype 1 and genotype 6, subtypes c to l [28], [30], [31], [32]. This explains why some patients, initially included in this trial as they were considered to be infected with HCV genotype 1, were in fact infected with HCV genotype 6.

Novel assays have been recently developed that aim at better discriminating among the different HCV genotype 1 subtypes and between genotypes 1 and 6. Abbott RealTi*m*e HCV Genotype II assay is a real-time PCR method using several sets of genotype- and subtype-specific primers and probes located in both the 5′NCR and the NS5B-coding region. As shown in Table 1, adding a second target region for analysis led to substantially improving HCV genotype 1 subtype identification compared to methods targeting the sole 5′NCR. However, in contrast with a previous report [33], we found that this assay failed to correctly identify HCV genotype 1 subtype in approximately 10% of cases. No obvious explanation was found when comparing the NS5B sequences of these strains with those that were correctly classified. However, in contrast with direct sequence analysis of the NS5B gene, which uses a long nucleotide sequence, the real-time PCR assay uses only the short sequence of its primers and probes. This probably explains that this assay may sometimes fail to differentiate HCV genotype 1 and several subtypes of genotype 6 (M. Bouvier-Alias, unpublished data). Improvement in the sequence of the primers and probes in order to correctly classify HCV subtypes 1a and 1b is underway in order to make this assay a useful tool for clinical trials and practice.

In the second-generation line probe assay, probes targeting the core-coding region were added to the probes targeting the 5′NCR already present in the first-generation assay, and a new PCR reaction was implemented to allow multiplex amplification of both regions. The goal was to improve discrimination between subtypes 1a and 1b and between genotypes 1 and 6. Better performance of INNO-LiPA HCV 2.0 than the first-generation assay has already been reported [30], [32]. We show here that, when excluding the small number of samples that could not be amplified with the PCR technique provided in the kit, correct identification of HCV subtypes 1a and 1b was achieved in more than 99% of cases. Therefore, INNO-LiPA HCV 2.0 currently is the best available commercial assay for HCV genotype 1 subtype identification and should be used in clinical trials and practice.

The ability of the tested assays to detect simultaneous infections by different HCV genotype 1 subtypes was not evaluated. However, the line probe assays have been shown to be more sensitive for the detection of multiple genotype infections than sequence-based assays, as they can detect minor viral populations representing more than 5%–10% of the total population [23]. This information is not yet available for the real-time PCR-based assay.

In conclusion, the choice of the target genome region, more than the technology, is crucial for HCV genotyping/subtyping. In the context of new HCV drug development, where correct HCV genotype 1 subtype determination is mandatory, HCV genotyping methods based on the exclusive analysis of the 5′NCR should be avoided because they poorly discriminate among these subtypes. The second-generation line probe assay is currently the best commercial assay for determination of HCV genotype 1 subtypes 1a and 1b. It can therefore be used locally in clinical trials to identify the HCV subtype and stratify the patients at inclusion, as well as to interpret efficacy and resistance data. When reporting final data, direct sequence analysis of the NS5B region and/or another coding region (for instance the region encoding the antiviral drug target HCV protein) should always be performed as it may identify mistyping or mis-subtyping with commercial assays, especially in the case of rare subtypes.

## Materials and Methods

### Materials

A total of 516 treatment-naïve patients infected with HCV genotype 1 included in a French multicenter clinical trial of pegylated IFN-α and ribavirin were studied. The characteristics of the patients have been previously described [26]. The protocol was approved by the local Ethics Committee (Comité Consultatif de Protection des Personnes dans la Recherche Biomédicale, CCPPRB) of the University Hospital of Nancy on October 24, 2000. All patients provided written informed consent. The study was conducted according to the guidelines of the Declaration of Helsinki, under provisions of Good Clinical Practices, or both. Data was collected by the study group, and analyzed by the authors with the help of the sponsor. The authors had unlimited access to the data and no limitation on publication was imposed by any party.

All patients had anti-HCV antibodies in serum, detectable serum HCV RNA (>600 IU/mL), increased alanine aminotransferase (ALT) levels on at least two determinations within the previous 6 months, liver biopsy findings consistent with chronic hepatitis C within 18 months before therapy, and compensated liver disease.

The clinical specimens used in the present study were the baseline serum samples from the 516 patients. The average HCV RNA level was 6.2±0.8 Log_10_ IU/mL and 70.2% of the samples had a high HCV RNA, >800,000 IU/mL (5.9 Log_10_ IU/mL) [26]. Sera were frozen at −70°C until use in this study.

### Study Design

The patients had been initially considered to be infected with HCV genotype 1 and included in the study based on a local determination of the HCV genotype and, eventually, subtype. In the present study, the exact genotype and subtype were determined by means of the reference method, i.e. direct sequence analysis of a portion of the nonstructural (NS) 5B-coding gene followed by phylogenetic analysis. The ability of the following commercial assays to correctly identify HCV subtypes 1a and 1b was assessed, in comparison with the reference method: i) an assay based on direct sequence analysis of the 5′NCR of HCV genome, Trugene HCV 5′NC Genotyping Kit (Siemens Medical Solutions Diagnostics, Tarrytown, New York); ii) the first-generation Line Probe Assay, INNO-LiPA HCV 1.0 (Innogenetics, Gent, Belgium), based on reverse-hybridization of PCR products using oligonucleotide probes in the 5′NCR; iii) the second-generation Line Probe Assay, INNO-LiPA HCV 2.0 (Innogenetics), based on reverse-hybridization of PCR products using oligonucleotide probes in the 5′NCR and the core-coding region; iv) A real-time PCR-based assay using primers and probes in both the 5′NCR and the NS5B-coding region, Abbott RealTi*m*e HCV Genotype II assay (Abbott Molecular, Des Plaines, Illinois).

### Direct Sequence Analysis of a Portion of the NS5B Gene Followed by Phylogenetic Analysis (Reference Method)

Briefly, total RNA was extracted from 200 µl of serum by using the High Pure Viral RNA kit (Roche Molecular Biochemicals, Mannheim, Germany), according to the manufacturer's instructions. The RNA pellet was eluted with 50 µl of diethyl pyrocarbonate-treated (DEPC) water and stored at −70°C until analysis. Complementary DNA (cDNA) synthesis was performed with 5 µl of total extracted RNA and 200 U of Superscript III reverse transcriptase (Invitrogen, Carlsbad, California). A nested PCR technique was used to amplify a DNA fragment located in the NS5B gene. The first round used external sense and antisense primers Sn755 and Asn1121 [34] and consisted of 35 PCR cycles at 95°C for 30 s, 58°C for 30 s, and 70°C for 30 s. The second round used internal sense primer NS5B–SI766 and antisense primer NS5B–ASI1110 [26] and consisted of 35 cycles at 95°C for 30 s, 58°C for 30 s, and 70°C for 30 s. PCR products were directly sequenced with the Big-Dye Terminator Cycle v3.1 sequencing kit on an ABI 3100 sequencer (Applied Biosystems, Foster City, California), according to the manufacturer's instructions. Phylogenetic analyses were carried out using genotypes 1 to 6 reference sequences available in GenBank, by means of the Phylogeny Inference Package (PHYLIP), version 3.65 [35]. Nucleotide sequences (286 bp) long were aligned with the reference sequences using CLUSTAL W [36]. Phylogenetic relationships were deduced by means of DNADIST-NEIGHBOR from PHYLIP. For neighbor-joining analysis, a Kimura 2-parameter distance matrix with a transition/transversion ratio (Ts/Tv) of 2.0 was used [37]. Phylogenetic trees were plotted with NJPlot and the figure was drawn with Dendroscope v2.3 (www.dendroscope.org) [38], [39]. Their robustness was assessed by bootstrap analysis of 1000 replicates by means of the SEQBOOT program from PHYLIP.

### Direct Sequence Analysis of the 5′NCR

For direct sequence analysis of the 5′NCR, The Trugene HCV 5′NC Genotyping Kit was used. This assay is based on semi-automated CLIP™ sequencing of the 5′ NCR. Briefly, total extracted RNA was used as a template for reverse transcription-PCR amplification in a single tube. A 244-bp fragment spanning nucleotide positions 68–311 in the 5′NCR (according to strain H77) was amplified. The reaction consisted of a “hot-start” protocol with an initial reverse transcription at 50°C for 30 min, a PCR activation step at 95°C for 15 min, 35 cycles at 95°C for 30 s, 55°C for 30 s, and 72°C for 1 min, and a final elongation at 72°C for 7 min. The amplified product was then labeled via the CLIP™ sequencing reaction. Briefly, bidirectional DNA sequencing was performed using two sequencing primers labeled with different fluorescent dyes, followed by electrophoresis and data analysis on the OpenGene® DNA sequencing system (Siemens Medical Solutions Diagnostics). For final interpretation, each bidirectional sequence was automatically aligned with a panel of more than 100 reference sequences with the GeneLibrarian™ module of the GeneObjects™ software (Siemens Medical Solutions Diagnostics), allowing genotype and subtype assignment based on percent sequence identity.

### Reverse Hybridization after PCR Amplification

The INNO-LiPA HCV 2.0 assay (Innogenetics) is provided with reagents for PCR amplification of two fragments spanning two thirds of the 5′NCR and a portion of the core-coding region, respectively. The products amplified with this method were used for hybridization with both INNO-LiPA HCV 1.0 and INNO-LiPA HCV 2.0 strips.

Briefly, reverse transcription-PCR was performed in a single tube. Twenty µl of extracted RNA was added to 30 µl of PCR master mix containing two pairs of biotinylated synthetic oligonucleotides corresponding to the two amplified regions. Reverse transcription was performed at 50°C for 30 min, followed by an initial PCR activation step of 95°C for 15 min. Then, 40 cycles of 95°C for 30 s, 50°C for 30 s, and 72°C for 15 s were carried out, with a final extension at 72°C for 2 min on a UNO-Thermoblock™ (Biometra, Goettingen, Germany). Two distinct biotinylated DNA fragments of 240 and 270 bp representing the 5′NCR and core-coding region, respectively, were produced.

After denaturation, the biotinylated PCR products were hybridized to oligonucleotide probes bound to the INNO-LiPA HCV 1.0 and INNO-LiPA HCV 2.0 nitrocellulose strip, respectively. INNO-LiPA HCV 1.0 contains two control lines and 19 5′NCR DNA probe lines specific for the different HCV genotypes and subtypes. INNO-LiPA HCV 2.0 contains the same lines, plus an additional core control line and three core DNA probe lines, specific for HCV genotype 1 subtypes and genotype 6. After the hybridization step, the unhybridized PCR product was washed from the strip, and alkaline phosphatase-labeled streptavidin (conjugate) was bound to the biotinylated hybrid. 5-bromo-4-chloro-3-indolylphosphate (BCIP)-nitroblue tetrazolium chromogen (substrate) reacts with the streptavidin-alkaline phosphate complex, forming a purple-brown precipitate, resulting in a visible banding pattern on the strip. AutoLiPA 2.0 (Innogenetics, Zwijndrecht, Belgium) was used to carry out hybridization and the developing color step.

### Real-Time PCR Determination of the HCV Genotype

The Abbott RealTi*m*e HCV Genotype II assay (Abbott Diagnostic) is based on real-time PCR amplification targeting two genome regions: the 5′NCR and the NS5B-coding region. The assay uses 4 primer pairs and the corresponding probes labeled with different fluorescent dyes: one pair targets the 5′NCR and is used for amplification of all HCV strains; the second and third pairs target the NS5B-coding region and are used to specifically amplify HCV subtype 1a and 1b strains, respectively; the fourth pair is used for amplification of the internal control. Briefly, HCV RNA was extracted from 200 µl of serum with the *m*2000_SP_ automated device (Abbott Molecular), according to the manufacturer's protocol. The PCR reactions were run in the *m*2000_RT_ automated device (Abbott Molecular). Three separate real-time PCR reactions (A, B and C) were run in parallel for each strain. The first step was reverse transcription by means of the *rTth* enzyme, followed by the PCR reaction that targets both 5′NCR and NS5B in reactions A and B, and only 5′NCR in reaction C. Probes labeled with different fluorophores were used in each PCR reaction to confirm the presence of HCV RNA; identify HCV subtype 1a and HCV genotype 3 (reaction A); identify HCV genotypes 1 and 2 and HCV subtype 1b (reaction B); identify HCV genotypes 4, 5 and 6 (reaction C). The assay was performed according to the manufacturer's instructions. Indeterminate results were retested from a serum volume of 500 µl.
